# Supplementary material for: Physiological, Photosynthetic Characteristic and Transcriptome Analysis of PsnWRKY70 Transgenic Populus simonii × Populus nigra Under Salt Stress
Source: Int J Mol Sci. 2024 Dec 25;26(1):81. doi: 10.3390/ijms26010081 (PMC11720120; doi:10.3390/ijms26010081)
Supplement: Supplementary file 1 [file ijms-26-00081-s001.zip › Table S1.pdf]

Table S1. The specific primers and reference genes that used in qRT-PCR verification.

Gene	Gene Classification	Forward primer (5′–3′)	Reverse primer (5′–3′)	Amplicon	
				L(bp)	Tm(°C)
Potri.016G137900	WRKY70	AACAAGCCAAGCAACTCCCT	ACCCCAGAAATCACATCGCC	197	60.0
Potri.019G067900	AAE6	GTGTATCTGTGGACCCTGCC	ATCGTAGGGGCTTCCACTCT	113	60.0
Potri.019G093400	CCD4	TATGGTCCCGTCCCTCCATT	AGGTGAGCCCCCTCCAAATA	195	60.0
Potri.009G044700	PRPA	GATGCGGCTAGGAGGGTTTT	TGCCTCTTTTGGCTTCCCAT	102	60.0
Potri.004G172300	CPD11	ATCACAAGGGAGCGTTGACT	TGGACACAACATCAGGGTGG	140	60.0
Potri.015G043400	GPSSS	GGAGCGGATTTTGAGGGTGA	TATCTCCTCCTCGGTTCCCC	196	60.0
At4g33380-like	Housekeeping	CTGCCTCTGCTGATACCT	GGAACCGAACCAATCTTCTC	272	60.0

AAE6, Probable acyl-activating enzyme 6; CCD4, Probable carotenoid cleavage dioxygenase 4; PRPA, Pentatricopeptide repeat-containing protein At2g29760; CPD11, Chaperone protein dnaJ 11; GPSSS, Heterodimeric geranylgeranyl pyrophosphate synthase small subunit. L, length of PCR production (in base pairs); Tm, melting temperature (in °C).
