# Supplementary material for: Physiological, Photosynthetic Characteristic and Transcriptome Analysis of PsnWRKY70 Transgenic Populus simonii × Populus nigra Under Salt Stress
Source: Int J Mol Sci. 2024 Dec 25;26(1):81. doi: 10.3390/ijms26010081 (PMC11720120; doi:10.3390/ijms26010081)
Supplement: Supplementary file 1 [file ijms-26-00081-s001.zip › Table S3-S6.pdf]

Table S3. GO enrichment of the DEGs between NT vs. OEX1 apical bud.

GO. ID	Description	Number in Input List	Number in BG/Ref.	*p*-Value	FDR
GO:0042592	homeostatic process	6	169	0.00021	0.014
GO:0019725	cellular homeostasis	6	168	0.00019	0.014
GO:0008152	metabolic process	110	11384	0.00014	0.014
GO:0055114	oxidation reduction	30	2000	9.2e-05	0.014
GO:0016491	oxidoreductase activity	36	2263	2.3e-06	0.00025
GO:0005506	iron ion binding	16	661	1.9e-06	0.00025
GO:0003824	catalytic activity	109	10587	4.7e-06	0.00034
GO:0020037	heme binding	13	596	0.00014	0.005
GO:0016705	oxidoreductase activity, acting on paired donors, with incorporation or reduction of molecular oxygen	14	661	0.00011	0.005
GO:0009055	electron carrier activity	15	738	0.00012	0.005
GO:0046906	tetrapyrrole binding	13	611	0.0002	0.0063
GO:0030246	carbohydrate binding	8	305	0.0006	0.017
GO:0016209	antioxidant activity	6	190	0.00073	0.018

Table S4. GO enrichment of the DEGs between NT vs. REX1 apical bud.

GO. ID	Description	Number in Input List	Number in BG/Ref.	*p*-Value	FDR
GO:0007018	microtubule-based movement	27	91	4.1e-72	2.3e-69
GO:0007017	microtubule-based process	28	144	1.2e-50	3.3e-48
GO:0006260	DNA replication	17	84	9.1e-32	1.7e-29
GO:0015995	chlorophyll biosynthetic process	6	14	5.1e-20	7e-18
GO:0006259	DNA metabolic process	27	329	1.9e-18	2.1e-16
GO:0015994	chlorophyll metabolic process	6	21	1.2e-14	1.1e-12
GO:0006779	porphyrin biosynthetic process	6	32	3.5e-10	2.8e-08
GO:0046148	pigment biosynthetic process	6	38	1.1e-08	7.5e-07
GO:0006778	porphyrin metabolic process	6	42	6.7e-08	4.1e-06
GO:0033014	tetrapyrrole biosynthetic process	6	43	1e-07	5.6e-06
GO:0042440	pigment metabolic process	6	48	6.2e-07	3.1e-05
GO:0033013	tetrapyrrole metabolic process	6	53	2.8e-06	0.00013
GO:0018130	heterocycle biosynthetic process	8	92	3.1e-06	0.00013
GO:0015979	photosynthesis	11	164	4.2e-06	0.00017
GO:0005976	polysaccharide metabolic process	12	231	0.00011	0.0042
GO:0051188	cofactor biosynthetic process	7	110	0.00074	0.024
GO:0006629	lipid metabolic process	26	801	0.0007	0.024
GO:0051186	cofactor metabolic process	9	169	0.00083	0.025
GO:0003777	microtubule motor activity	27	91	4.1e-72	1.9e-69
GO:0003774	motor activity	27	107	1.8e-62	4.2e-60
GO:0051002	ligase activity, forming nitrogen-metal bonds	6	14	5.1e-20	4.6e-18
GO:0051003	ligase activity, forming nitrogen-metal bonds, forming coordination complexes	6	14	5.1e-20	4.6e-18
GO:0016851	magnesium chelatase activity	6	14	5.1e-20	4.6e-18
GO:0004097	catechol oxidase activity	6	17	2.6e-17	1.7e-15
GO:0016682	oxidoreductase activity, acting on diphenols and related substances as donors, oxygen as acceptor	6	17	2.6e-17	1.7e-15
GO:0016818	hydrolase activity, acting on acid anhydrides, in phosphorus-containing anhydrides	47	919	1e-15	5.8e-14
GO:0016462	pyrophosphatase activity	46	893	1.4e-15	7e-14

GO:0017111	nucleoside-triphosphatase activity	45	872	2.7e-15	1.2e-13
GO:0016817	hydrolase activity, acting on acid anhydrides	47	944	5.1e-15	2.1e-13
GO:0003887	DNA-directed DNA polymerase activity	6	25	1.2e-12	4.5e-11
GO:0034061	DNA polymerase activity	6	26	3.1e-12	1.1e-10
GO:0016679	oxidoreductase activity, acting on diphenols and related substances as donors	6	29	4e-11	1.3e-09
GO:0003824	catalytic activity	229	10587	1.6e-08	4.9e-07
GO:0016787	hydrolase activity	89	3150	6.3e-08	1.8e-06
GO:0005524	ATP binding	86	3601	0.00025	0.0063
GO:0032559	adenyl ribonucleotide binding	86	3601	0.00025	0.0063
GO:0001882	nucleoside binding	87	3826	0.0013	0.032
GO:0003854	3-beta-hydroxy-delta5-steroid dehydrogenase activity	7	120	0.0018	0.036
GO:0030554	adenyl nucleotide binding	86	3816	0.002	0.036
GO:0033764	steroid dehydrogenase activity, acting on the CH-OH group of donors, NAD or NADP as acceptor	7	120	0.0018	0.036
GO:0016491	oxidoreductase activity	56	2263	0.0019	0.036
GO:0001883	purine nucleoside binding	86	3816	0.002	0.036
GO:0016229	steroid dehydrogenase activity	7	120	0.0018	0.036

Table S5. GO enrichment of the DEGs between NT vs. OEX1 fifth functional leaf.

GO. ID	Description	Number in Input List	Number in BG/Ref.	*p*-Value	FDR
GO:0009073	aromatic amino acid family biosynthetic process	10	21	1.3e-26	4.9e-24
GO:0046417	chorismate metabolic process	10	21	1.3e-26	4.9e-24
GO:0009072	aromatic amino acid family metabolic process	11	30	5.1e-24	1.3e-21
GO:0019438	aromatic compound biosynthetic process	11	41	2.2e-18	4.3e-16
GO:0043648	dicarboxylic acid metabolic process	11	49	1.4e-15	2.1e-13
GO:0016053	organic acid biosynthetic process	21	228	1.4e-10	1.6e-08
GO:0046394	carboxylic acid biosynthetic process	21	228	1.4e-10	1.6e-08
GO:0044283	small molecule biosynthetic process	25	320	8.4e-10	8.2e-08
GO:0006725	cellular aromatic compound metabolic process	12	97	4.4e-09	3.8e-07
GO:0006633	fatty acid biosynthetic process	11	94	7.6e-08	5.6e-06
GO:0006631	fatty acid metabolic process	12	110	7.9e-08	5.6e-06
GO:0044281	small molecule metabolic process	50	1080	6.6e-07	4.2e-05
GO:0032787	monocarboxylic acid metabolic process	13	142	9.2e-07	5.5e-05
GO:0016052	carbohydrate catabolic process	12	141	1e-05	0.00055
GO:0043436	oxoacid metabolic process	27	499	1.2e-05	0.00057
GO:0006082	organic acid metabolic process	27	500	1.3e-05	0.00057
GO:0019752	carboxylic acid metabolic process	27	499	1.2e-05	0.00057
GO:0042180	cellular ketone metabolic process	27	501	1.3e-05	0.00057
GO:0009309	amine biosynthetic process	11	138	7.2e-05	0.0029
GO:0008652	cellular amino acid biosynthetic process	10	124	0.00015	0.0057
GO:0044275	cellular carbohydrate catabolic process	9	106	0.00019	0.0066
GO:0046164	alcohol catabolic process	9	106	0.00019	0.0066
GO:0008610	lipid biosynthetic process	20	369	0.0002	0.0068
GO:0019320	hexose catabolic process	8	97	0.00069	0.02
GO:0006007	glucose catabolic process	8	97	0.00069	0.02
GO:0046365	monosaccharide catabolic process	8	97	0.00069	0.02
GO:0006629	lipid metabolic process	33	801	0.001	0.03
GO:0006096	glycolysis	6	62	0.0011	0.031
GO:0044282	small molecule catabolic process	9	127	0.0016	0.042
GO:0003824	catalytic activity	308	10587	1.2e-08	5.8e-06
GO:0016840	carbon-nitrogen lyase activity	5	21	2.1e-07	5.3e-05
GO:0005506	iron ion binding	32	661	3.3e-05	0.0055
GO:0016765	transferase activity, transferring alkyl or aryl (other	7	62	5.6e-05	0.007

	than methyl) groups				
GO:0043492	ATPase activity, coupled to movement of substances	11	150	0.00024	0.02
GO:0042626	ATPase activity, coupled to transmembrane movement of substances	11	150	0.00024	0.02
GO:0016820	hydrolase activity, acting on acid anhydrides, catalyzing transmembrane movement of substances	11	159	0.00051	0.037

Table S6. GO enrichment of the DEGs between NT vs. REX1 fifth functional leaf.

GO. ID	Description	Number in Input List	Number in BG/Ref.	*p*-Value	FDR
GO:0006629	lipid metabolic process	22	801	2.3e-06	0.00098
GO:0006073	cellular glucan metabolic process	6	110	5e-05	0.0073
GO:0044042	glucan metabolic process	6	110	5e-05	0.0073
GO:0015979	photosynthesis	7	164	0.00022	0.024
GO:0006633	fatty acid biosynthetic process	5	94	0.00039	0.034
GO:0044283	small molecule biosynthetic process	10	320	0.00056	0.04
GO:0016053	organic acid biosynthetic process	8	228	0.00074	0.04
GO:0046394	carboxylic acid biosynthetic process	8	228	0.00074	0.04
GO:0032787	monocarboxylic acid metabolic process	6	142	0.00088	0.042
